# Phospho-HDAC6 Gathers Into Protein Aggregates in Parkinson’s Disease and Atypical Parkinsonisms

**DOI:** 10.3389/fnins.2020.00624

**Published:** 2020-06-23

**Authors:** Samanta Mazzetti, Mara De Leonardis, Gloria Gagliardi, Alessandra Maria Calogero, Milo Jarno Basellini, Laura Madaschi, Ilaria Costa, Francesca Cacciatore, Sonia Spinello, Manuela Bramerio, Roberto Cilia, Chiara Rolando, Giorgio Giaccone, Gianni Pezzoli, Graziella Cappelletti

**Affiliations:** ^1^Department of Biosciences, Università degli Studi di Milano, Milan, Italy; ^2^Fondazione Grigioni per il Morbo di Parkinson, Milan, Italy; ^3^UNITECH NO LIMITS, Università degli Studi di Milano, Milan, Italy; ^4^Imaging TDU, IFOM Foundation, The FIRC Institute of Molecular Oncology, Milan, Italy; ^5^Unit of Neuropathology and Neurology, Fondazione IRCCS Istituto Neurologico Carlo Besta, Milan, Italy; ^6^S. C. Divisione Oncologia Falck and S. C. Divisione Anatomia Patologica, Ospedale Niguarda Ca’ Granda, Milan, Italy; ^7^Parkinson Institute, ASST “G.Pini-CTO,” Milan, Italy; ^8^Center of Excellence on Neurodegenerative Diseases, Università degli Studi di Milano, Milan, Italy

**Keywords:** HDAC6, phosphorylated-HDCA6, protein aggregation, α-synuclein, tau, Parkinson’s disease, parkinsonism

## Abstract

HDAC6 is a unique histone deacetylase that targets cytoplasmic non-histone proteins and has a specific ubiquitin-binding activity. Both of these activities are required for HDAC6-mediated formation of aggresomes, which contain misfolded proteins that will ultimately be degraded via autophagy. HDAC6 deacetylase activity is increased following phosphorylation on serine 22 (phospho-HDAC6). In human, HDAC6 localizes in neuronal Lewy bodies in Parkinson’s disease (PD) and in oligodendrocytic Papp–Lantos bodies in multiple system atrophy (MSA). However, the expression of phospho-HDAC6 in *post-mortem* human brains is currently unexplored. Here, we evaluate and compare the distribution of HDAC6 and its phosphorylated form in human brains obtained from patients affected by three forms of parkinsonism: two synucleinopathies (PD and MSA) and a tauopathy (progressive supranuclear palsy, PSP). We find that both HDAC6 and its phosphorylated form localize with pathological protein aggregates, including α-synuclein-positive Lewy bodies in PD and Papp–Lantos bodies in MSA, and phospho-tau-positive neurofibrillary tangles in PSP. We further find a direct interaction of HDAC6 with α-synuclein with proximity ligation assay (PLA) in neuronal cell of PD patients. Taken together, our findings suggest that both HDAC6 and phospho-HDAC6 regulate the homeostasis of intra-neuronal proteins in parkinsonism.

## Introduction

Histone deacetylases (HDACs) belong to a family of enzymes closely associated with gene expression and chromatin dynamics. Among them, HDAC6 is a well-characterized class II HDAC showing particular structural features and a number of domains that interact with specific, non-histone substrates (Valenzuela-Fernández et al., 2008). HDAC6 functions are mediated by two catalytic deacetylase domains (DD1 and DD2) and a C-terminal zinc finger ubiquitin-binding domain, which allows HDAC6 to bind mono- and poly-ubiquitin chains with high affinity (Hook et al., 2002; Boyault et al., 2006). HDAC6 cytoplasmic localization is regulated by a SE14 domain (Bertos et al., 2004) and a nuclear export signal (NES), which enables HDAC6 to accumulate in the cytoplasm (Verdel et al., 2000). HDAC6 dynein-binding domain (DMB) enables the dynein-mediated transport of ubiquitinated proteins along microtubules (Richter-Landsberg and Leyk, 2013). The distinct functions of HDAC6 rely on its deacetylase and ubiquitin-binding activity.

In the cytoplasm, HDAC6 deacetylase activity targets mainly non-histone proteins (Richter-Landsberg and Leyk, 2013). Moreover, HDAC6 is the major microtubule-associated deacetylase responsible for microtubule acetylation (Hubbert et al., 2002). Alpha-tubulin acetylation of lysine 40 only occurs on polymerized microtubules (Matsuyama et al., 2002). While still controversial, there is evidence that HDAC6 preferentially acts on free tubulin dimer (Matsuyama et al., 2002; Skultetyova et al., 2017). An increase in microtubule acetylation stimulates anterograde and retrograde transport processes via kinesin-1 and dynein recruitment (Dompierre et al., 2007). Nevertheless, the HDAC6 deacetylase function is inhibited by tau (Perez et al., 2009). Tau is sensitive to acetylation, and its hyper-acetylation leads to fibrillization *in vitro* and also microtubule assembly impairment. Interestingly, tau itself was identified as one of the HDAC6 targets (Cohen et al., 2011). Importantly, HDAC6 deacetylase activity is increased after serine 22 phosphorylation by glycogen synthase kinase 3β (Chen et al., 2010).

HDAC6 ubiquitin-binding activity is well characterized in neurodegenerative disorders, including Parkinson’s disease (PD), Alzheimer’s disease (AD), and Huntington’s disease (Simões-Pires et al., 2013). The formation of intracellular aggregates containing misfolded proteins is a cellular hallmark of several neurodegenerative diseases. In eukaryotes, ubiquitin-proteasome system (UPS) and autophagy are the two main degradation pathways that clear misfolded proteins. A close relationship exists between the UPS and autophagy, since autophagy may act as a compensatory mechanism in case of UPS impairment (Pandey et al., 2007). In neurodegenerative disorders, many ubiquitinated proteins accumulate, leading to saturation of UPS (Thibaudeau et al., 2018). In this context, HDAC6 acts as a cellular stress sensor and coordinates cell responses to fight accumulation of cytotoxic protein aggregates. Thus, HDAC6 interacts with dynein and promotes the retrograde transport of misfolded proteins to aggresome (Kawaguchi et al., 2003). Aggresome is a cytosolic structure enriched in polyubiquitin, γ-tubulin, acetylated α-tubulin, HDAC6, and misfolded proteins like α-synuclein (Richter-Landsberg and Leyk, 2013). Aggresome formation has been linked to the biogenesis of inclusion bodies, such as Lewy bodies in PD (Ardley et al., 2003; Kawaguchi et al., 2003) and Papp–Lantos bodies in multiple system atrophy (MSA) (Chiba et al., 2012). In addition, HDAC6 is involved in autophagy where it regulates autophagosome maturation and fusion with the lysosome (Richter-Landsberg and Leyk, 2013) through the activation following deacetylation of the actin-remodeling factor cortactin. This event causes the local assembly of a microfilament network that enhances fusion activity and induces protein aggregate degradation (Lee and Yao, 2010). HDAC6 also interacts with tau through its SE14-domain and not with its ubiquitin binding domain (Ding et al., 2008). This interaction leads to the accumulation and colocalization of HDAC6 and tau in the perinuclear region in an aggresome-like formation, especially when proteasome is inhibited.

Here, we unraveled the distribution of HDAC6 and its phosphorylated form in *post-mortem* human brains of PD and primary atypical parkinsonism patients. Specifically, here we: (i) evaluated the distribution of HDAC6 and its phosphorylated form, phospho-HDAC6, in PD and atypical parkinsonisms, such as MSA and PSP; (ii) analyzed the colocalization of phospho-HDAC6 with α-synuclein and phospho-tau; and (iii) checked for the interaction between phospho-HDAC6/HDAC6 and α-synuclein in PD.

## Materials and Methods

### Human Brain Tissue

*Post-mortem* human brains obtained from patients fulfilling clinical and neuropathological diagnostic criteria for PD (*n* = 5; Braak stage VI of synuclein pathology) (Alafuzoff et al., 2009), MSA (*n* = 1), PSP (*n* = 2), AD (*n* = 2), and from control subjects (*n* = 4) were used (Supplementary Table S1; Nervous Tissues Bank of Milan). Written informed consent was obtained from all subjects in compliance with relevant laws and institutional guidelines and approved by the appropriate institutional committees. All the patients were enrolled and followed during their disease by neurologists experienced in movement disorders and dementia at Parkinson’s Centre ASST G. Pini-CTO of Milan. The clinical diagnosis of PD was established according to the UK Brain Bank criteria (Hughes et al., 1992, 2001). The clinical diagnosis was confirmed by neuropathological analysis carried out according to the current BrainNet Europe Consortium guidelines (Alafuzoff et al., 2009; Dickson and Weller, 2011) by two experts (GGi and MB). Control subjects were clinically free from neurological diseases. Brains were fixed in 10% buffered formalin for at least 1 month at 20°C. After dehydration and clearing steps, selected areas (medulla, mesencephalon, and entorhinal cortex) were embedded in paraffin, and 5 μm thick sections were cut and processed for the following analysis.

### Immunofluorescence

Medulla, mesencephalon, and entorhinal cortex paraffin-embedded sections were dewaxed in xylene and rehydrated. Antigens were retrieved using 80% formic acid for 20 min at room temperature. Before primary antibody incubation, samples were incubated for 20 min with 1% BSA diluted in 0.01 M phosphate saline buffer (PBS) containing 0.1% Triton X-100 (PBS-T). Primary antibodies (rabbit anti-HDAC6, 1:50, GeneTex or rabbit anti-phospho-HDAC6, 1:50, GeneTex and mouse anti-α-synuclein LB509, 1:500, Abcam or mouse anti-phospho-tau AT8, 1:150, Thermo Fisher Scientific or mouse anti-β-amyloid, 1:2000, Wako) in 1% BSA diluted in PBS-T were incubated overnight at room temperature. After washing, samples were incubated for 2 h at room temperature with highly pre-adsorbed secondary antibodies, in particular Alexa Fluor^®^ 568 goat anti-mouse (Molecular Probes) and Alexa Fluor^®^ 488 donkey anti-rabbit (Molecular Probes). TO-PRO^®^-3 (1:1000 for 10 min; Molecular Probes) was used for nuclei counterstaining. Finally, samples were mounted using 0.01 M PBS-glycerol (1:2) and examined with a TCS SP8 confocal microscope Leica equipped with an Argon laser coupled with a hybrid detector, a diode-pumped solid-state laser coupled with a photomultiplier tube and a helium/neon mixed gas laser coupled with a hybrid detector.

### Scan Acquisition and Colocalization Analysis

In order to obtain images representative of the whole thickness of the samples, immunostained slices were acquired using the Hamamatsu Nanozoomer S60 scanner (Nikon) equipped with an Olympus 20×/0.75 PlanSApo objective (Olympus), a linear ORCA-Flash 4.0 digital CMOS camera (Hamamatsu), a fluorescence imaging module equipped with a L11600 mercury lamp (Hamamatsu), and two six-position filter wheels for excitation and emission. The excitation filters used were 387/11 (DAPI), 480/17 (FITC), and 556/20 (TRITC). The quantitative analysis of the acquired immunofluorescence images was performed using ImageJ software (NIH). The areas analyzed (dorsal motor nucleus of vagus, reticular nucleus, inferior olivary nucleus, *substantia nigra*, red nucleus, and entorhinal cortex) were previously selected using region of interest (ROI) manager to produce an accurate ROI for the subsequent analysis. The colocalization of α-synuclein and phospho-tau with phospho-HDAC6 was determined by measuring the Manders’ coefficients and Pearson’s correlation coefficient (Bolte and Cordelières, 2006).

### Proximity Ligation Assay (PLA)

The *in situ* proximity ligation assay (PLA) enables the detection of protein–protein interactions in intact tissues (Söderberg et al., 2006; Longhena et al., 2018). For PLA procedure, we analyzed human paraffin-embedded brain sections from the *substantia nigra* of PD patients and controls using the Duolink assay kit (Sigma Aldrich) according to the manufacturer’s instructions. Briefly, after dehydration, samples were treated as described for immunohistochemistry (formic acid, BSA/PBS-T as blocking solution) and incubated with the same mix of primary antibodies (rabbit anti-HDAC6/mouse anti-α-synuclein LB509 and rabbit anti-phospho-HDAC6/mouse anti-α-synuclein LB509). After washing, samples were incubated with the goat anti-mouse IgG and anti-rabbit IgG secondary antibodies conjugated, respectively, with Duolink^®^ PLA MINUS and PLUS oligonucleotides in Duolink^®^ antibody diluent for 2 h at 37°C. Samples were then treated with a solution of Duolink^®^ ligation solution (1:5) and Duolink^®^ ligase (1:40) at 37°C for 1 h. After that, samples were incubated with a solution of Duolink^®^ amplification reagent green 1:5 and Duolink^®^ polymerase (1:80) at 37°C for 2.5 h. Finally, TO-PRO^®^-3 was used for nuclei counterstaining. The samples were mounted using Mowiol^®^(Calbiochem)-DABCO (Sigma). PLA-labeled samples were examined with a TCS SP8 confocal microscope. In order to analyze PLA puncta/neuron, we acquired four images (20× magnification) for each PD patient and control (PLA HDAC6/α-synuclein: PD *n* = 3, controls *n* = 3; PLA p-HDAC6/α-synuclein: PD *n* = 4, controls *n* = 4). PLA puncta were quantified in the neuronal cell bodies containing neuromelanin, as revealed by phase contrast microscopy, using ImageJ (Cell Counter plugin).

### Statistical Analysis

Statistical comparisons were conducted by the Mann-Whitney (control subjects *n* = 4; PD patients *n* = 4) or Student’s *t*-test for PLA puncta/neuron. Statistical significance was assessed using GraphPad Prism software. Significance was established at *p* < 0.05.

## Results

### Phospho-HDAC6 Localizes in Lewy Bodies of PD Patients

**FIGURE 1:**
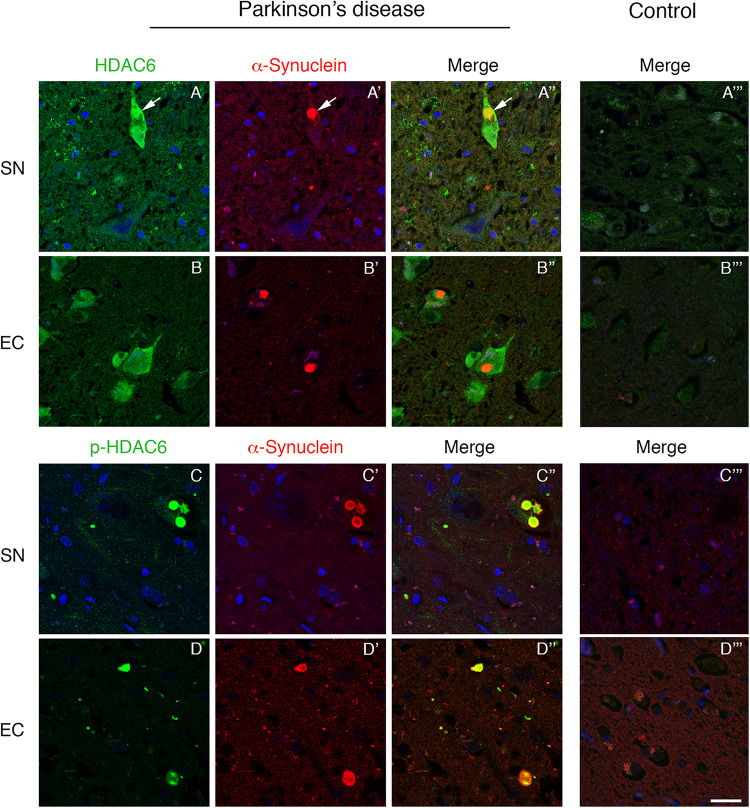
Lewy bodies contain both HDAC6 and phospho-HDAC6 in *substantia nigra* and entorhinal cortex of PD patients. Confocal microscopy analysis shows that HDAC6 staining [green in **(A,A”,B,B”)**] is abundant in neuronal cell bodies which present α-synuclein positive Lewy bodies [red in **(A’,A”,B’,B”)**; white arrows]. Notably, in entorhinal cortex, HDAC6 staining is also present in the majority of neurons. In control subjects **(A”’,B”’)**, HDAC6 staining is point and weak in the cytoplasm. Phospho-HDAC6 [green in **(C,C”,D,D”)**] is present as punctate staining in neuronal cell bodies, and it accumulates into α-synuclein positive Lewy bodies [red in **(C’,C”,D’,D”)**], while it is almost undetectable in neuronal cell bodies in controls **(C”’,D”’)**. Nuclei were counterstained using TOPRO-3 (blue). Scale bar, 25 μm.

To investigate the relationship between HDAC6 and α-synuclein, we performed double immunofluorescence on PD patient brain. We initially focused on the *substantia nigra* that is the first brain region directly linked to the motor symptoms during PD progression. We found that HDAC6 is highly expressed in the *substantia nigra* neurons that contain α-synuclein positive Lewy bodies. Interestingly, HDAC6 strongly colocalized with Lewy bodies (Figures 1A–A”), while in control subjects the HDAC6 staining is weak and diffuse in the cytoplasm (Figure 1A”’). The same expression pattern was observed in other brain areas involved in PD progression, including the entorhinal cortex (Figures 1B–B”), the dorsal motor nucleus of vagus (Supplementary Figures S1A–A”) and the *locus coeruleus* (Supplementary Figures S1B–B”). In the entorhinal cortex, HDAC6 expression was not restricted to cells containing Lewy bodies, but it was present in the majority of neurons (Figures 1B–B”) while it is almost undetectable in controls (Figure 1B”’).

We evaluated for the first time the distribution of phospho-HDAC6 in α-synuclein inclusion bodies, and we performed double immunofluorescence for α-synuclein and phospho-HDAC6 in PD and control brains. In the *substantia nigra*, phospho-HDAC6 was almost undetectable in neuronal cell bodies of control subjects (Figures 1C”’,D”’), while it showed a neuronal punctate staining in PD brains where it mainly colocalized with α-synuclein-positive Lewy bodies (Figures 1C–C”). In addition, phospho-HDAC6 staining appeared scattered in the neuropil. The same distribution was observed in the dorsal motor nucleus of vagus (Supplementary Figures S1C–C”) and *locus coeruleus* (Supplementary Figures S1D–D”). Notably, cortical Lewy bodies also contained phospho-HDAC6, as shown in entorhinal cortex (Figures 1D–D”).

### Phospho-HDAC6 Accumulates in Papp–Lantos Bodies of an MSA Patient

**FIGURE 2:**
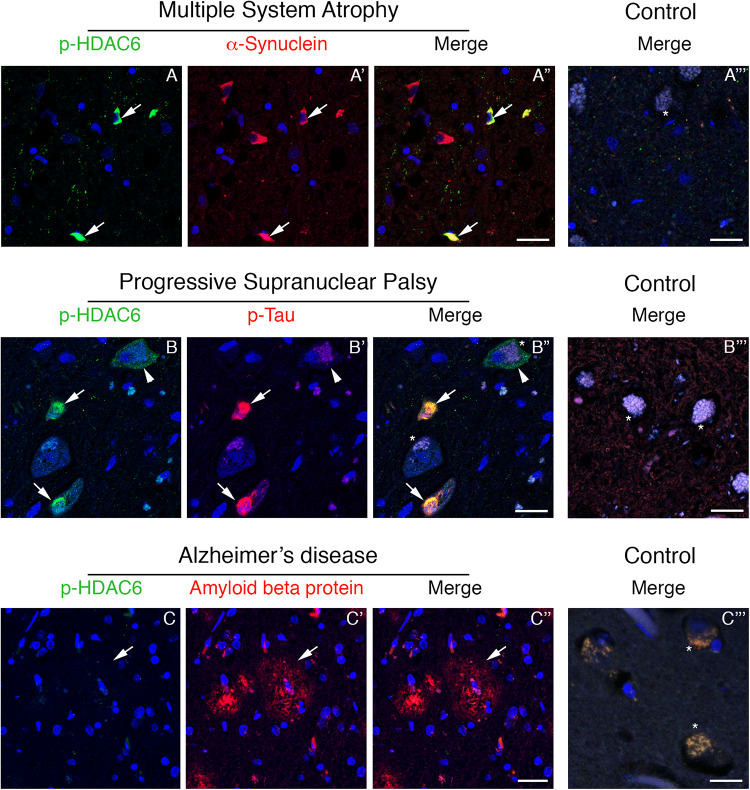
Phospho-HDAC6 gathers into protein aggregates in inferior olivary nucleus of MSA and PSP, but not in entorhinal cortex of AD patients. In MSA **(A–A”)**, numerous Papp–Lantos inclusions in oligodendrocytes, which are positive for α-synuclein (red), show phospho-HDAC6 staining (green, white arrows). In PSP patients **(B–B”)**, phospho-HDAC6 (green) is present as punctate staining in neuronal cell bodies, (white arrowhead). Neurons also present aggregates of phospho-tau (visualized using AT8 antibody, red) known as neurofibrillary tangles **(B’,B”)**, which resulted to be positive for phospho-HDAC6 (green) as indicated by white arrows. Phospho-HDAC6 staining was absent in amyloid beta protein positive plaques in entorhinal cortex in AD patients **(C–C”)**. In controls **(A”’,B”’,C”’)**, phospho-HDAC6 is negative in both neuronal (visible for the presence of lipofuscin; asterisks) and glial cell bodies and the neuropil is poorly stained. α-Synuclein is present only with its characteristic synaptic pattern **(A”’)**. Phospho-tau and amyloid beta protein are absent in controls, respectively, in the inferior olivary nucleus **(B”’)** and in entorhinal cortex **(C”’)**. Samples were counterstained using TOPRO-3 (blue). Scale bar, 25 μm.

To test whether phosphorylated HDAC6 accumulation in Lewy bodies is restricted to PD, we evaluated the expression of phospho-HDAC6 in other parkinsonisms. We performed double immunofluorescence for α-synuclein and phospho-HDAC6 on one patient affected by MSA, a synucleinopathy characterized by the presence of α-synuclein aggregates mainly in oligodendrocytes. We focused on inferior olivary nucleus (Figure 2) and putamen (not shown), two regions enriched in white matter and therefore in oligodendrocytes. In controls, phospho-HDAC6 was absent in neuronal cell bodies and in all the glial cells (Figure 2A”’), while it was present in neuropil. In an MSA patient, oligodendrocytes showed the typical triangular or flame-shaped α-synuclein aggregates, known as Papp–Lantos inclusions (Figure 2A’). Double immunostaining for phospho-HDAC6 and α-synuclein revealed that some oligodendrocytes contained both inclusion bodies of α-synuclein and intense phospho-HDAC6 staining (Figure 2A”). On the contrary, in the neuronal cell bodies of the inferior olivary nucleus, phospho-HDAC6 was absent (Figure 2A). All together, these data suggest that phospho-HDAC6 colocalizes with α-synuclein aggregates not only in PD but also in MSA.

### Phospho-HDAC6 and Phospho-Tau Colocalize in Inferior Olivary Nucleus in PSP

Since phospho-HDAC6 is present in α-synuclein inclusion bodies of PD and also in the MSA patient, we asked whether phospho-HDAC6 could also be linked to protein aggregation in neurodegenerative diseases beyond synucleinopathies.

Phospho-tau is the main component of neurofibrillary tangles, pathological hallmarks of tauopathies (Dickson and Weller, 2011). To test whether phospho-HDAC6 localized in neurofibrillary tangles, we performed double immunofluorescence for phospho-HDAC6 and phospho-tau in atypical parkinsonism PSP. Histopathological analysis confirmed that α-synuclein aggregates were absent in the brain of PSP patients (data not shown). We analyzed the inferior olivary nucleus where neurofibrillary tangles accumulate in PSP brain (Dickson and Weller, 2011). We found an intense and diffuse phospho-HDAC6 staining in neuronal cell bodies and within PSP neurofibrillary tangles, which displayed strong phospho-tau staining (Figures 2B–B”), whereas controls were negative (Figure 2B”’).

Phospho-HDAC6 consistently localized with cytoplasmic protein aggregates in PD, MSA, and PSP. To evaluate whether phospho-HDAC6 identified both intra- and extra-neuronal protein aggregates in neurodegenerative diseases, we analyzed AD human brains. Interestingly, phospho-HDAC6 staining was absent in extra-neuronal amyloid beta plaques in entorhinal cortex (Figures 2C–C”) as well as in controls (Figure 2C”’). All together, these results indicate that phospho-HDAC6 identifies exclusively intracellular protein aggregates.

### Specific Distribution of Phospho-HDAC6 in Parkinsonisms

**FIGURE 3:**
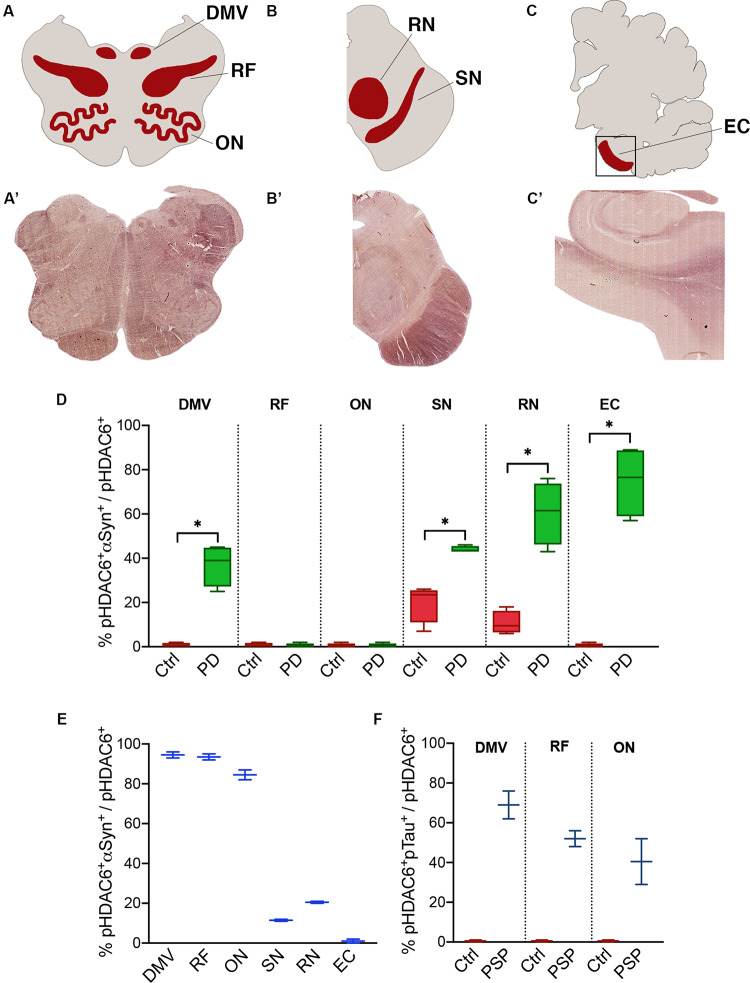
Fraction of phospho-HDAC6 colocalizing with α-synuclein or phospho-tau. Areas that underwent quantification procedure are highlighted in **(A–C)** (red areas), which stand for a schematic representation of, respectively, **(A’–C’)** (brightfield images obtained from scanner acquisition). Colocalization rates are evaluated as M1 Mander’s coefficient between phospho-HDAC6 and α-synuclein in PD compared to control **(D)** and in MSA **(E)** or phospho-tau in PSP **(F)**. Values are expressed as intervals (indicated in the Supplementary Table S2) that consider inter-individual variability. DMV, dorsal motor nucleus of vagus; EC, entorhinal cortex; ON, olivary nucleus; pHDAC6, phospho-HDAC6; pTau, phospho-tau; RF, reticular formation; RN, red nucleus; SN, *substantia nigra*. Mann-Whitney test *p < 0.05.

To investigate phospo-HDAC6 accumulation and protein aggregation in parkinsonism, we extended our analysis to the medulla, midbrain, and cortex of PD, MSA, and PSP patients (Figures 3A–C’ and Supplementary Figure S2).

We evaluated the fraction of phospho-HDAC6 colocalizing with α-synuclein in PD and MSA brains. Phospho-HDAC6 and α-synuclein significantly colocalized in the dorsal motor nucleus of vagus, *substantia nigra*, red nucleus, and entorhinal cortex in PD compared to controls (Figure 3D). These brain regions are heavily affected by α-synuclein pathology at the analyzed time point (Braak stage VI) (Dickson and Weller, 2011). On the contrary, phospho-HDAC6 and α-synuclein did not colocalize in the reticular formation and in the inferior olivary nucleus that is widely unaffected in PD patients.

MSA neurodegeneration largely affects highly myelinated regions (Dickson and Weller, 2011). In line with this, we analyzed samples from one MSA patient and found high colocalization of phospho-HDAC6 and α-synuclein in inferior olivary nucleus, dorsal motor nucleus of vagus, and reticular formation (Figure 3E). On the contrary, in the *substantia nigra* of the MSA patient, phospho-HDAC6/α-synuclein colocalization was not different from control (Figure 3E vs. 3D), this can be explained with the advanced neurodegenerative state of this region at the final stage of the disease. Interestingly, phospho-HDAC6 and α-synuclein colocalization was undetectable in entorhinal cortex of MSA and control subjects (Figure 3E).

The analysis of the two PSP patients suggests an increase in the colocalization of phospho-HDAC6 and phospho-tau in the dorsal motor nucleus of vagus, inferior olivary nucleus, and reticular formation (Figure 3F). We also evaluated the fraction of α-synuclein and phospho-tau that colocalized with phospho-HDAC6 in the brain regions previously analyzed. Overall, we found that α-synuclein extensively colocalized with phospho-HDAC6 in PD (Manders’ coefficient: 72–99%) and to a less extent in the MSA patient (Manders’ coefficient: 43–70%). Furthermore, phospho-tau widely colocalized with phospho-HDAC6 in PSP (Manders’ coefficient: 60–87%). Mander’s colocalization analysis is also supported by Pearson’s coefficient study (Supplementary Table S3).

### HDAC6 and Phospho-HDAC6 Interact With α-Synuclein in PD

**FIGURE 4:**
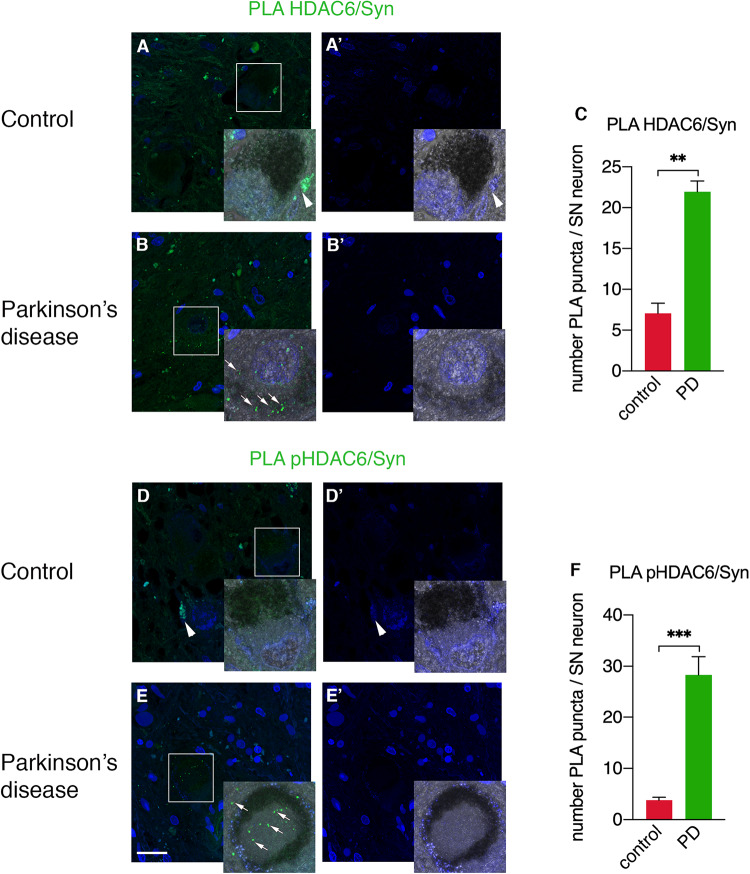
Confocal analysis of PLA for HDAC6 and α-synuclein and for phospho-HDAC6 and α-synuclein in *substantia nigra* in control and PD patients. PLA signal (green) for both HDAC6/α-synuclein and phospho-HDAC6/α-synuclein is present as punctate diffuse staining within neuronal cell bodies in PD patients **(B,E)** as indicated by white arrows, while it is absent or scarce in controls **(A,D)**. Magnification, 1.25×. Samples were counterstained using TOPRO-3 (blue). In **(A’,B’,D’,E’)**, the images show the blue channel; besides nuclei staining, there is evident tissue autofluorescence (arrowheads), which enables the exclusion of aspecific staining and to identify the specific PLA signal (arrows). **(C,F)** Quantifications of PLA puncta/SN neuron containing neuromelanin identifiable with the phase contrast superimposed image ± SEM (*n* = 3–4 per groups; analyzed neurons: *n* = 18–44 for groups; **p = 0.01; ***p = 0.001). Scale bar, 25 μm.

To unravel the direct interactions between HDAC6 and cellular aggregate components, we employed a PLA approach to detect HDAC6/α-synuclein and phospho-HDAC6/α-synuclein interactions in *substantia nigra* neurons of PD patients and controls. While HDAC6/α-synuclein PLA signal was undetectable or scarce in neuromelanin-containing cell bodies in control brains (Figures 4A,A’,C), it showed a scattered profile in the neuropil and a significantly increased punctate staining within neuronal cell bodies in PD brain (Figures 4B,B’,C). Interestingly, also phospho-HDAC6/α-synuclein PLA signal was undetectable or scarce in controls (Figures 4D,D’,F), whereas it was significantly increased in *substantia nigra* neurons of PD (Figures 4E,E’,F). These data clearly indicate the direct interaction of HDAC6 and phospho-HDAC6 with α-synuclein in PD brain.

HDAC6 localization in aggresome hinges on retrograde transport (Kawaguchi et al., 2003). We showed here that HDAC6 and phospho-HDAC6 interact with α-synuclein and we therefore tested the involvement of phospho-HDAC6 in the retrograde transport of α-synuclein. We evaluated the expression of α-synuclein, phospho-HDAC6, and the motor protein dynein in control (Supplementary Figure S3A–A”’) and PD (Supplementary Figure S3B–B”’) brains and found that phospho-HDAC6 colocalization with α-synuclein and dynein significantly increased in PD patients (Supplementary Figure S3C), thus suggesting trafficking alterations.

## Discussion

Despite intra- and extra-neuronal protein aggregates are hallmarks of many neurodegenerative disorders, their exact composition and the molecular mechanisms leading to their formation are still unclear. In this study, we investigated the role of HDAC6 and its phosphorylated version in PD and in atypical parkinsonism MSA and PSP. Besides confirming the presence of HDAC6 in PD and in the MSA patient, we showed for the first time that phospho-HDAC6 localizes in Lewy bodies where it interacts with α-synuclein in PD.

We compared the expression of phospho-HDAC6 in PD, the synucleinopathy MSA, and the tauopathy PSP. Interestingly, phospho-HDAC6 is expressed in Papp–Lantos bodies in the MSA patient and in neurofibrillary tangles of PSP patients, while it was absent in extra-neuronal amyloid beta plaques in AD brains. Despite the limitation of a small patient cohort and the need of confirmation in future larger studies, all the subjects included in this article have been accurately checked for both clinical diagnosis and neuropathological analysis. On this basis, our results suggest that phospho-HDAC6 could be a common hallmark in the formation of intracellular protein aggregates in different forms of parkinsonisms.

α-Synuclein is the main component of the typical inclusion bodies that are linked to neurodegeneration in synucleinopathies, such as PD and MSA (Dickson et al., 2009; Goedert et al., 2017). The identification of α-synuclein interacting partners has a major implications for elucidating the neurodegenerative process (Yan et al., 2018). We therefore investigated the interplay of α-synuclein with HDAC6 and its phosphorylated active version phospho-HDAC6 (Chen et al., 2010). To date, the data available on HDAC6 expression in *post-mortem* PD patient brain indicate that it accumulates into Lewy bodies in cerebral cortex (Kawaguchi et al., 2003). Here, we first confirmed that HDAC6 gathers into α-synuclein-positive Lewy bodies in cerebral cortex. We also extended the analysis to neuromelanin-containing neurons in *substantia nigra*, *locus coeruleus*, and dorsal motor nucleus of vagus in *post-mortem* PD brain. In all these regions, HDAC6 was expressed in Lewy bodies, and it is therefore a hallmark for brain areas affected by α-synuclein pathology.

Further, we evaluated the expression of phospho-HDAC6 in PD and found that is restricted to Lewy bodies. In addition, PLA experiments showed that both HDAC6 and phospho-HDAC6 directly interacted with α-synuclein. This close interaction strongly suggests the importance of HDAC6 and phospho-HDAC6 in protein aggregation during neurodegeneration.

There is evidence about the involvement of HDAC6 in cellular management of misfolded protein and macroautophagy (reviewed in Richter-Landsberg and Leyk, 2013; Simões-Pires et al., 2013; Yan, 2014). *In vitro* experiments demonstrate that HDAC6 promotes the retrograde transport of polyubiquitinated misfolded proteins to the aggresome, via the interaction with dynein motor. Moreover, HDAC6 knock-down impairs aggresome formation and clearance of misfolded protein aggregates from the cytoplasm (Kawaguchi et al., 2003). Therefore, HDAC6 can support proper protein homeostasis and be neuroprotective (Zheng et al., 2013; Bourque et al., 2014; Du et al., 2014). The role of HDAC6 in neurodegeneration is still controversial. In animal experimental models of PD and Huntington’s diseases, HDAC6 inhibition rescues axonal transport defects and is beneficial for the neuronal survival (Dompierre et al., 2007; Godena et al., 2014; Pinho et al., 2016; Jian et al., 2017). HDAC6 inhibition also provides protection against oxidative stress on neurons *in vitro* (Leyk et al., 2017). On the other hand, HDAC6 knock-down impairs aggresome formation and clearance of misfolded protein aggregates causing cell death *in vitro* (Kawaguchi et al., 2003). Therefore, HDAC6 could support proper protein homeostasis and be neuroprotective (Zheng et al., 2013; Bourque et al., 2014; Du et al., 2014). To shed light on this complex mechanism, we addressed the role of HDAC6 and its phosphorylation *ex vivo* in human brain samples. In line with HDAC6 beneficial role, our current data indicate that HDAC6 could have a protective function during neurodegeneration. Indeed, we found an increase of HDAC6 and phospo-HDAC6 in PD, MSA, and PSP patients. Considering that HDAC6 phosphorylation increases its deacetylase activity (Chen et al., 2010) that is implicated in aggresome formation (Kawaguchi et al., 2003), increased HDAC6 levels could be the results of a compensatory mechanism for misfolded protein degradation.

Neurodegenerative diseases are characterized by specific types of protein aggregates. For example, MSA is a synucleinopathy characterized by α-synuclein aggregation in oligodendrocytes. PSP is a tauopathy where phospho-tau aggregates in neurons, while amyloid beta plaques in AD exist as extracellular aggregates (Dickson and Weller, 2011). We found that phospho-HDAC6 colocalized with α-synuclein in MSA and phospho-tau in PSP but not with extra-neuronal amyloid aggregates in AD. These data come from few patients and we are aware that need to be replicated in a bigger cohort. However, this restricted expression pattern could indicate that phospho-HDAC6 accumulation is not a ubiquitous marker of protein aggregates. To note, phospho-HDAC6 selectively identifies cells in disease-specific manner (glia in MSA and neurons in PSP). Taken together, our results suggest that HDAC6 phosphorylation is specifically involved in pathological and intra-cellular protein aggregation occurring in parkinsonisms. While MSA-related Papp–Lantos inclusions are HDAC6 positive (Miki et al., 2011), here we provide a comprehensive analysis of phospho-HDAC6 distribution in several brain areas of the MSA patient and the two PSP patients. We also determined the fraction of phospho-HDAC6 colocalizing with α-synuclein or phospho-tau. We found area- and disease-specific changes in colocalization between phospho-HDAC6 and α-synuclein. In particular, the olivary nucleus, a region in which Lewy bodies are absent in PD, lacks phospho-HDAC6 positive α-synuclein aggregates in control and PD subjects. On the contrary, the same region that is affected in MSA phospho-HDAC6 and α-synuclein heavily colocalize in Papp–Lantos bodies.

Several lines of evidence support the idea that many points of convergence exist among parkinsonisms. For example, α-synuclein and tau aggregations co-occur in a spectrum of neurodegenerative disorders (Lippa et al., 1998; Hamilton, 2000; Judkins et al., 2002; Mori et al., 2002; Yancopoulou et al., 2005). Another interesting point linking α-synuclein to tau is that mutations in the *SNCA* and *MAPT* genes can both lead to neurodegenerative disorders characterized by parkinsonism (Polymeropoulos et al., 1997; Dumanchin et al., 1998; Spillantini et al., 1998; Fujioka et al., 2014). In addition, we propose that HDAC6 could be considered as a common hallmark of parkinsonisms, independently from the nature of protein aggregates (Lewy bodies, Papp–Lantos bodies, and neurofibrillary tangles) and affected cells (neurons and glia). HDAC6 and phospho-HDAC6 gather into protein aggregates in PD, MSA, and PSP. Moreover, HDAC6 was already been established as a partner of tau in AD (Ding et al., 2008; Cook et al., 2012), and, interestingly, tau protein interacts and regulates the activity of HDAC6 (Simões-Pires et al., 2013).

Further analysis will aim to provide new insights into the complex role of the HDAC6 phosphorylation in controlling neurodegeneration in PD and parkinsonism.

## Data Availability Statement

The datasets generated for this study are available on request to the corresponding author.

## Ethics Statement

The studies involving human participants were reviewed and approved by Ethics Committees of Ospedale Niguarda Ca’ Granda, Milan, Italy and Fondazione IRCCS Istituto Neurologico Carlo Besta, Milan, Italy. Written informed consent for participation was not required for this study in accordance with the national legislation and the institutional requirements. Informed consent for the donation of tissue to the brain bank was obtained from all patients.

## Author Contributions

SM, GP, and GC made substantial contributions to the conception and design of the work. SM, GGa, MD, AC, MJB, LM, IC, FC, SS, MB, and GC made the acquisition, analysis, and interpretation of data for the work. SM, MD, AC, RC, CR, GGi, GP, and GC drafted the work and revised it critically for important intellectual content. All authors contributed to the final approval of the version to be published.

## Conflict of Interest

The authors declare that the research was conducted in the absence of any commercial or financial relationships that could be construed as a potential conflict of interest.
