# Supplementary material for: Phospho-HDAC6 Gathers Into Protein Aggregates in Parkinson’s Disease and Atypical Parkinsonisms
Source: Front Neurosci. 2020 Jun 23;14:624. doi: 10.3389/fnins.2020.00624 (PMC7324673; doi:10.3389/fnins.2020.00624)
Supplement: Supplementary file 1 [file Data_Sheet_1.PDF]

Supplementary Material

1 Supplementary Data

5 µm mesencephalic brain sections from both healthy controls and PD patients were deparaffinized and submitted to triple immununofluorescence with anti-α- Synuclein antibody (1:500; Abcam, sheep, which recognizes a sequence near the C-terminus of human α-Synuclein,), anti-Dynein antibody (1:40; Millipore, clone 74.1, mouse) and phospho-HDAC6 (1:50). After the rehydration procedure samples were pre-treated in 80% formic acid, the sections were then incubated with: i) a blocking solution (1% bovine serum albumin, BSA, in 0.01 M PBS plus 0.1% Triton X-100, 20 minutes, room temperature), ii) a mixture of primary antibodies containing diluted 1:100 for 2 hours at 37°C with 1% BSA in 0.01M PBS plus 0.1% Triton X-100, then overnight at room temperature. For the indirect immunofluorescence procedure, antigens were revealed with the secondary donkey anti-sheep antibodies conjugated to Alexa Fluor®-568 (Molecular Probes), donkey anti-mouse antibodies conjugated to Alexa Fluor®-488 (Molecular Probes) and donkey anti-rabbit antibodies conjugated to Alexa Fluor®-633 (Molecular Probes), diluted respectively 1:200 and 1:100 in 0.1% BSA in 0.01 M PBS (2 hours, 37°C). Finally, sections were counterstained with DAPI diluted and mounted with PBS plus glycerol (dilution ratio 1:2).

2 Supplementary Figures and Tables

Supplementary Table 1

Profiles for the subjects included in the present study.

Diagnosis	Gender	Age at onset	Age at death	Disease duration (year)
**CTRL**	M	/	71	/
**CTRL**	F	/	93	/
**CTRL**	M	/	70	/
**CTRL**	F	/	64	/
**PD**	M	57	75	18
**PD**	F	59	79	20
**PD**	M	59	75	16
**PD**	M	62	73	11
**PD**	M	40	59	19
**MSA-C**	F	56	62	6
**PSP**	M	72	79	7
**PSP**	F	63	72	9
**AD**	F	45	59	14
**AD**	M	n.a.	75	n.a.

Abbreviations: n.a.: data not available

Supplementary Table 2

Fraction of phospho-HDAC6 colocalizing with α-Synuclein or phospho-Tau (Mander's coefficient, M1).

Area	Control (n=4) pHDCA6/ α-Synuclein	PD (n=4) pHDCA6/ α-Synuclein	MSA (n=1) pHDCA6/ α-Synuclein	Control (n=3) pHDCA6/ pTau	PSP (n=2) pHDCA6/ pTau
Substantia nigra	7-26%	43-46%	12%	n.a.	n.a.
Red nucleus	6-18%	43-76%	20%	n.a.	n.a.
Olivary nucleus	0-2%	0-2%	82-87%	0	30%-52%
DMV	0-2%	25-45%	93-96%	0	62-76%
Reticular nucleus	0-2%	0-2%	93-94%	0	48-56%
Entorhinal cortex	0-2%	57-89%	0	n.a.	n.a.

Abbreviation: DMV = dorsal motor nucleus of vagus; MSA: Multiple System Atrophy; n.a.: data not available; pHDAC6: phospho-HDAC6; PD: Parkinson's disease; PSP: Progressive Supranuclear Palsy; pTau: phospho-Tau.

Supplementary Table 3

Pearson's correlation coefficient for phospho-HDAC6 with α-Synuclein or phospho-Tau. Pearson's correlation is positive, so it was expressed as a percentage to be comparable with Mander's coefficient.

Area	Control (n=4) pHDCA6 α-Synuclein	PD (n=4) pHDCA6 α-Synuclein	MSA (n=1) pHDCA6 α-Synuclein	Control (n=3) pHDCA6 pTau	PSP (n=2) pHDCA6 pTau
Substantia nigra	5-27%	37-51%	5%	n.a.	n.a.
Red nucleus	6-18%	43-76%	12%	n.a.	n.a.
Olivary nucleus	0-2%	0-2%	43-45%	0	35%-58%
DMV	0-2%	25-45%	45-51%	0	56-71%
Reticular nucleus	0-2%	0-2%	41-45%	0	43-45%
Entorhinal cortex	0-2%	57-89%	0	n.a.	n.a.

Abbreviation: DMV = dorsal motor nucleus of vagus; MSA: Multiple System Atrophy; n.a.: data not available; pHDAC6: phospho-HDAC6; PD: Parkinson's disease; PSP: Progressive Supranuclear Palsy; pTau: phospho-Tau.

Supplementary Figure 1

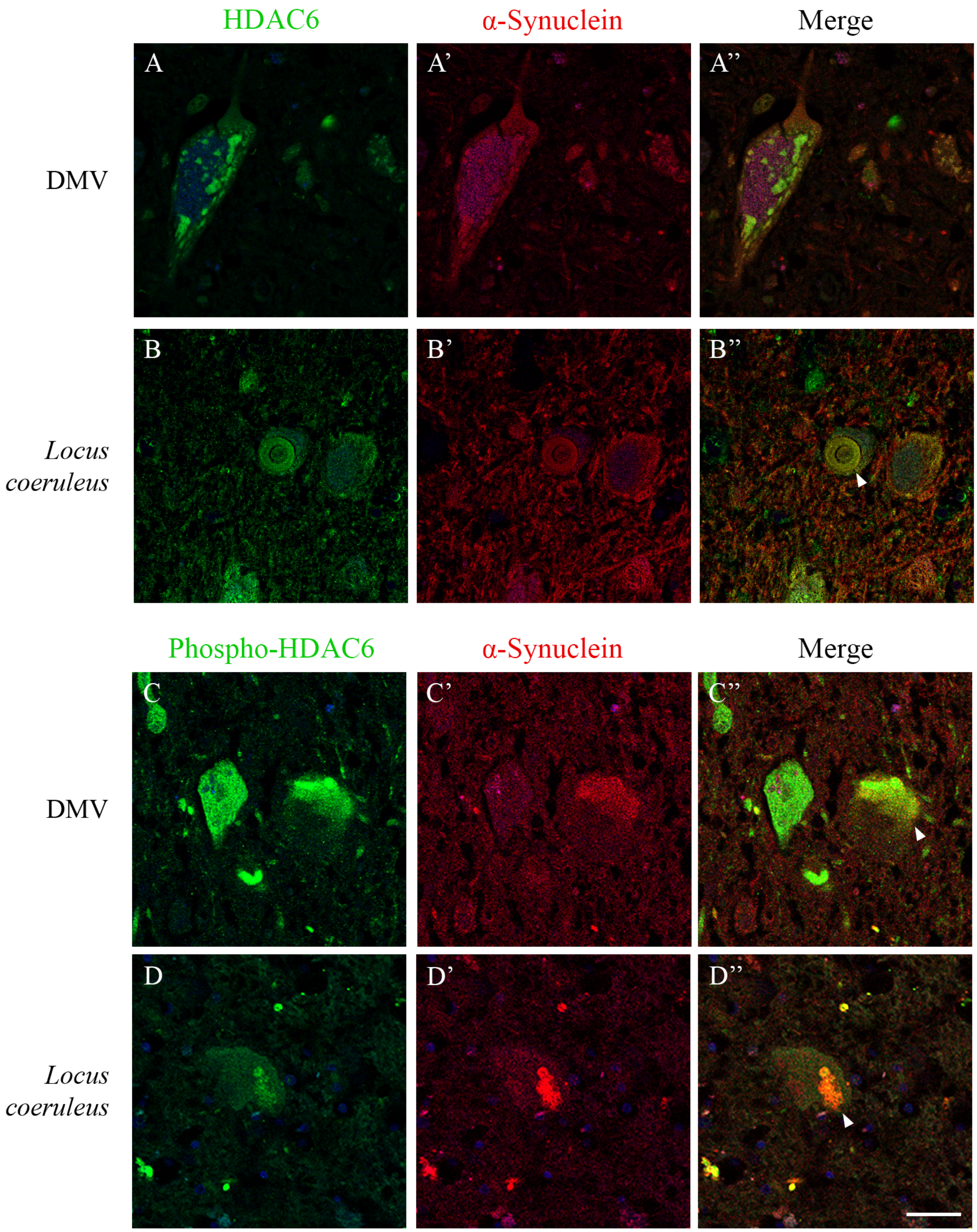

Supplementary Figure 1. Distribution of HDAC6 and phospho-HDAC6 in DMV and *locus coeruleus* in PD. HDAC6 and phospho-HDAC6 staining are visible in Lewy bodies (arrowheads). Scale bar, 25 µm. DMV = dorsal motor nucleus of vagus.

Supplementary Figure 2

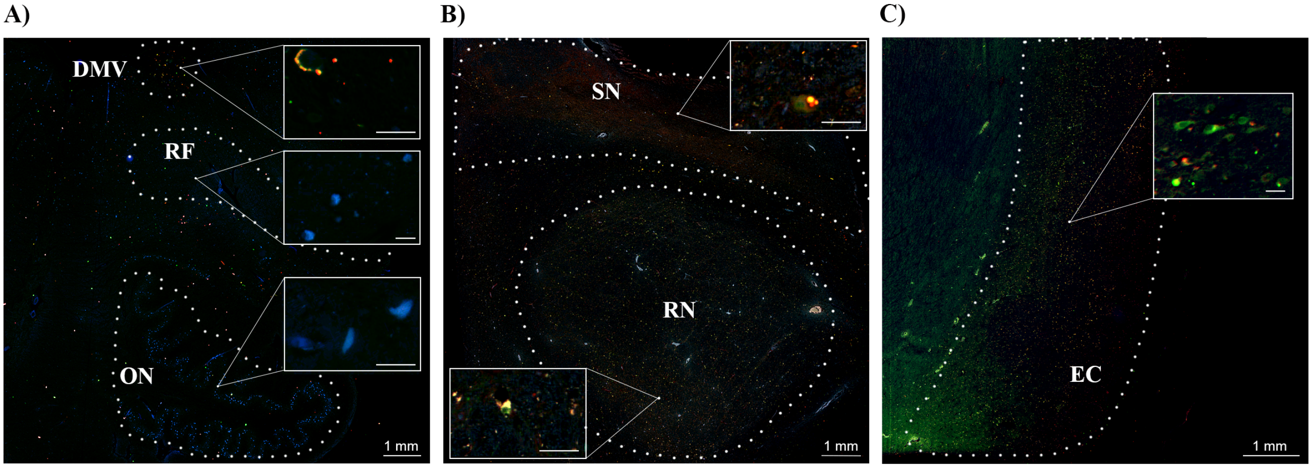

Supplementary Figure 2: Representative images for α-Synuclein and phospho-HDAC6 of the areas analysed in Figure 3. α-Synuclein (red signal) phospho-HDAC6 (green signal) staining is shown in medulla (A), mesencephalon (B) and entorhinal cortex (C) are shown. The blue signal is due to lipofuscin. The quantitative analysis (Mander's coefficient) was performed at the inset magnification. Scale bar, 100 µm.

Supplementary Figure 3

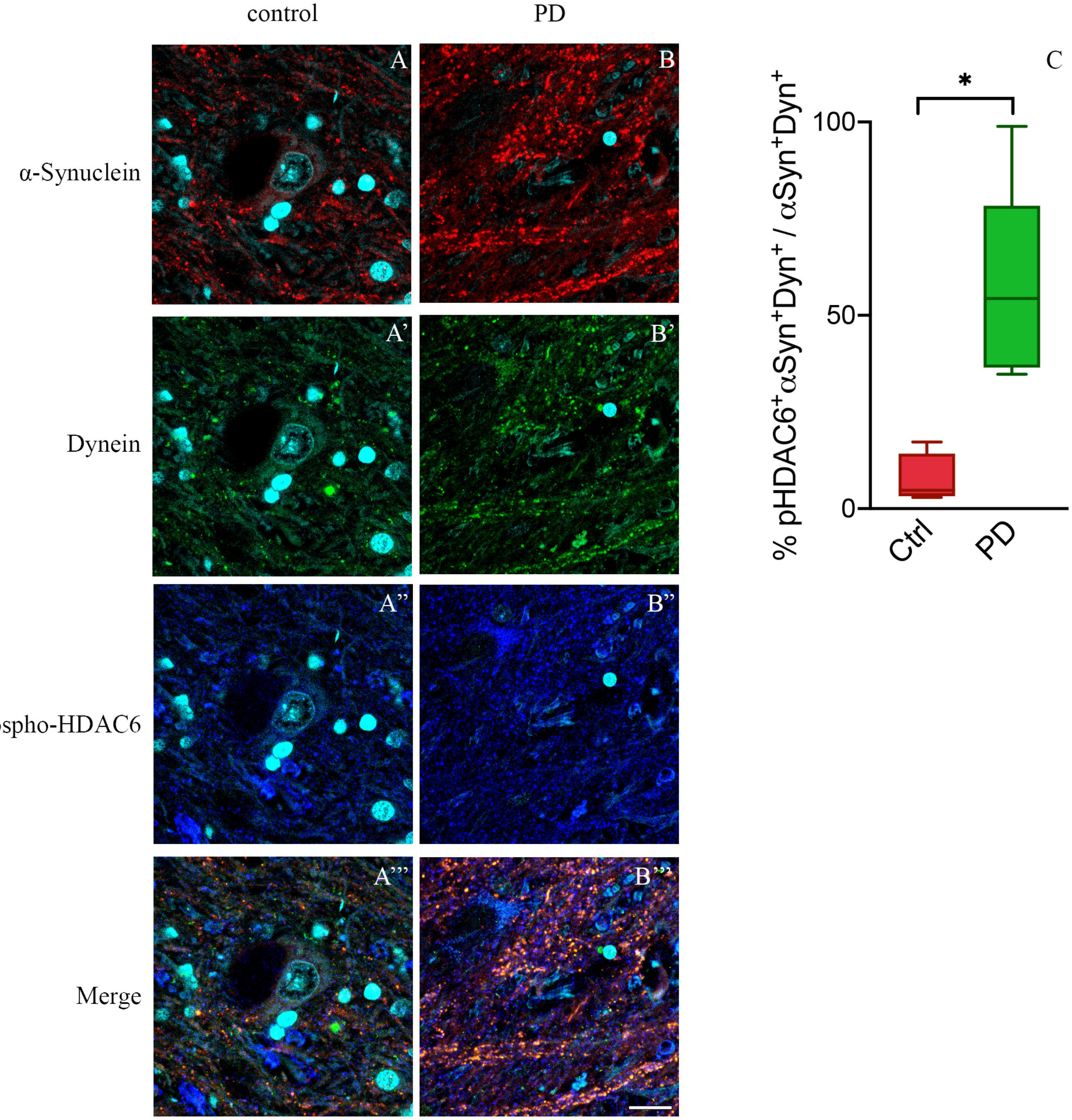

Supplementary Figure 3: Confocal analysis of triple staining for Dynein, α-Synuclein and phospho-HDAC6 in control and PD in *substantia nigra.* Compared to controls (A-A'''), PD sample (B-B''') display a significant increase of phospho-HDAC6 staining, which overlaps with both α-Synuclein and Dynein signal (C). Mann-Whitney test *$p<0.05$ (4 control subjects and 4 PD patients). Scale bar, 25 µm.
